# Narrow Escape: A Novel Approach to the Endovascular Treatment of Superior Vena Cava Syndrome Secondary to Pacemaker Leads with Excellent Long-term Outcomes

**DOI:** 10.7759/cureus.7249

**Published:** 2020-03-12

**Authors:** Yury Malyshev, Sergey Ayzenberg, Sonu Sahni, Mazin Khalid, Jeffrey Le

**Affiliations:** 1 Cardiology, Maimonides Medical Center, Brooklyn, USA; 2 Internal Medicine, Brookdale University Hospital Medical Center, Brooklyn, USA; 3 Research Medicine, New York Institute of Technology College of Osteopathic Medicine, New York, USA; 4 Primary Care, Touro College of Osteopathic Medicine, New York, USA

**Keywords:** pacemaker complication, lead complication, interventional cardiology, balloon angioplasty, superior vena cava (svc) syndrome, superior vena cava (svc) obstruction, permanent pacemaker (ppm) complication

## Abstract

Pacemaker or defibrillator placement is a common procedure done in more and more patients due to increased longevity and the prominence of cardiac disease. With more indications for cardiac implantable electrode devices, the devices themselves have evolved into more complex structures with more leads. The mechanical stress, risk of infection, and decreased blood flow through the superior vena cava (SVC) put patients at risk for SVC obstruction. Herein, we present a rare case of complete SVC obstruction secondary to fibrosis due to pacemaker leads which was treated with venoplasty and showed excellent long-term results. We also review the current literature on different approaches to treating SVC obstruction in this group of patients.

## Introduction

Superior vena cava syndrome (SVCS) is a result of obstructed blood flow through the superior vena cava (SVC), causing characteristic symptoms. Historically, the most common etiology of SVCS is malignancy [1]. However, since the invention and more widespread use of permanent pacemakers (PPMs) and implantable cardioverter-defibrillators (ICDs), pacing and defibrillator leads are becoming a more common cause of SVCS. Despite this being a growing concern for patients with PPMs or ICDs, the optimal treatment strategy is not known. Herein, we present the case of a patient with symptomatic SVC obstruction caused by a pacemaker lead. All of the patient’s symptoms were relieved after the venoplasty procedure, and he has been symptom-free for seven years without angiographic evidence of SVC restenosis. We also review the current understanding of the etiology of SVCS and treatment options.

## Case presentation

A 75-year-old male, with a history of coronary artery disease post-coronary artery bypass grafting (CABG) in 1990, peripheral vascular disease, hypertension, hyperlipidemia, and sinus node dysfunction with a dual-chamber pacemaker placed in 1998 and generator change in 2004, presented in 2011 with facial swelling, distention of the veins of the neck and chest, and redness of the face and neck. His clinical presentation was consistent with SVCS. The initial blood work was not significant (Table 1).

**Table 1:** Laboratory Studies BUN: blood urea nitrogen; Ca: calcium; Hgb: hemoglobin; INR: international normalized ratio; K: potassium; LDL: low-density lipoprotein; Na: sodium; PTT: partial thromboplastin time; WBC: white blood cells

Name	Value
Creatinine, serum (mg/dL)	1.2
BUN (mg/dL)	13
Na (mmol/L)	139
K (mmol/L)	4.3
Ca (mg/dL)	9.4
LDL (mg/dL)	62
WBC (K/UL)	6.6
Hgb (g/dL)	14.9
Platelets (K/UL)	155
INR	1.1
PTT (sec)	36

**Figure 1:**
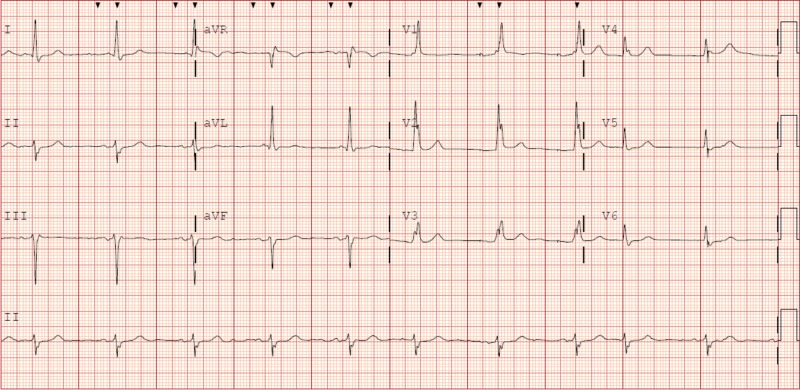
Electrocardiogram of the patient with atrial paced rhythm, right bundle branch block (RBBB), left anterior fascicular block (LAFB), and left ventricular hypertrophy (LVH)

An electrocardiogram (EKG) showed atrial paced rhythm with a right bundle branch block (RBBB), left anterior fascicular block (LAFB), and left ventricular hypertrophy (LVH) (Figure 1).

**Figure 2:**
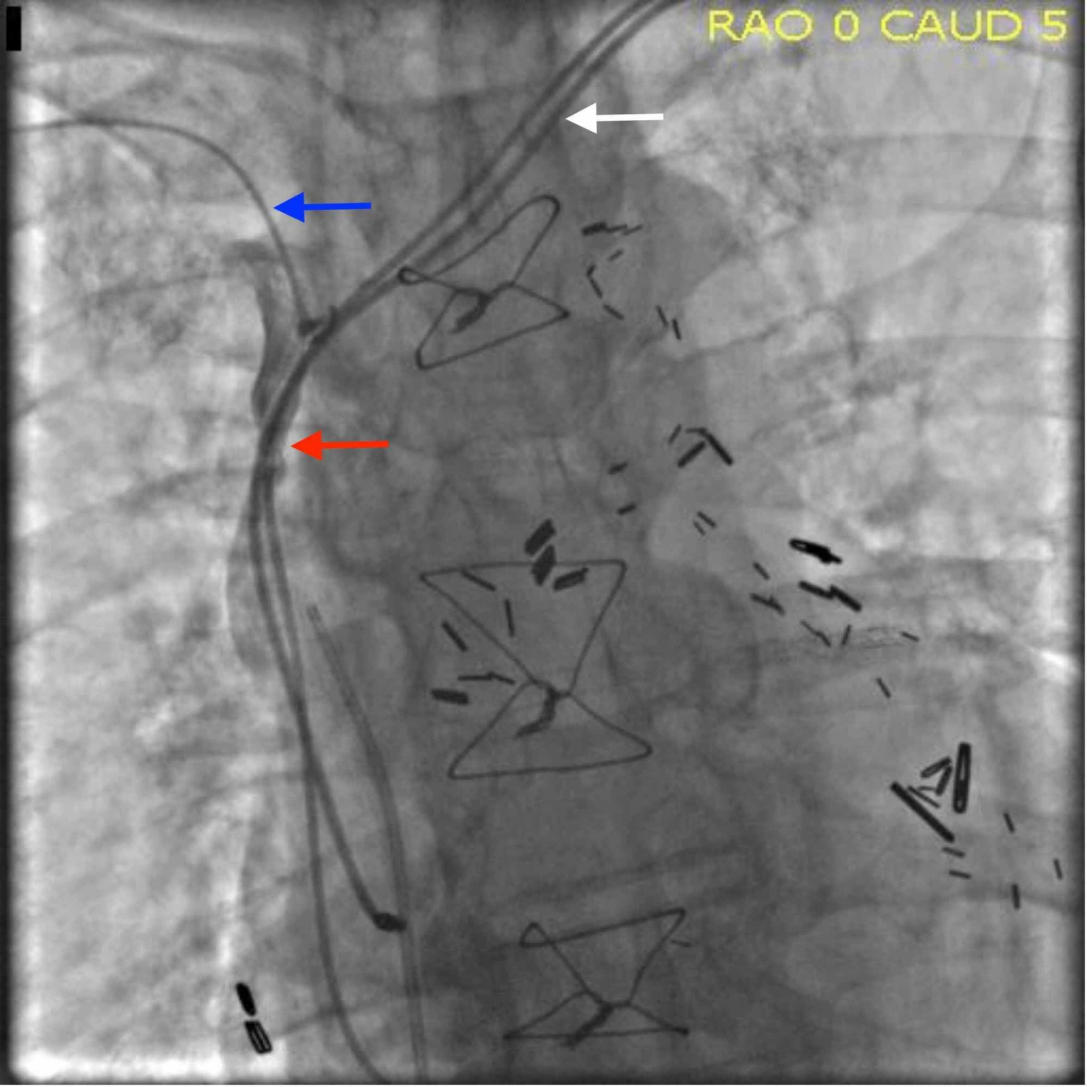
Superior vena cava (SVC) obstruction before intervention The angiogram shows complete occlusion of the SVC (red arrow), the Terumo Glidewire (blue arrow), and the pacemaker leads (white arrow).

The patient was taken to the catheterization lab where a 6-French Terumo sheath (Terumo Interventional Systems, Somerset, NJ) was placed in the right femoral vein and 4-French Terumo sheath in the right brachial vein. An angiogram confirmed the complete occlusion of the SVC caused by the pacemaker wires (Figure 2). The lesion could not be crossed via the femoral approach because the occlusion was so severe. A 4-French Terumo multipurpose A (MPA) 2 catheter was introduced via the right brachial vein and a Terumo Glidewire® (Terumo Interventional Systems, Somerset, NJ) was then used to cross the total occlusion. A 6-French Terumo sheath in the femoral vein was then exchanged for the 7-French Terumo sheath. The Terumo sheath and a 20 mm x 120 cm Amplatz Goose Neck™ snare (Medtronic, Inc., Minneapolis, MN) were used to retrieve the wire from below.

**Figure 3:**
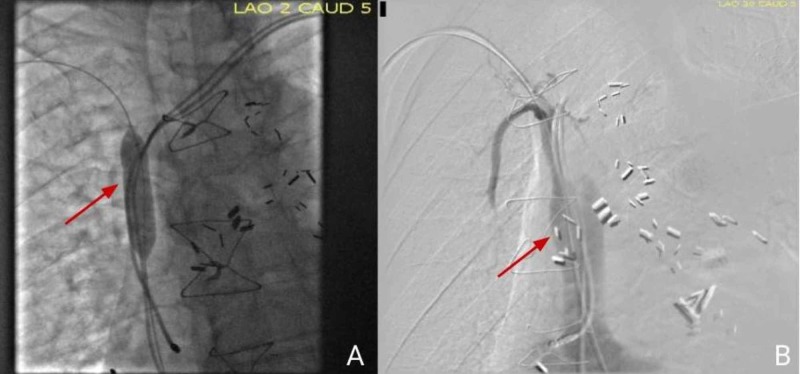
Images post-venoplasty A) Balloon dilatation (red arrow); B) cineangiography confirmed that superior vena cava is widely patent (red arrow)

Venoplasty, using 12 mm x 40 mm EverCross™ OTW PTA dilatation catheter (Medtronic, Inc., Minneapolis, MN), was used to perform three inflations to a pressure of 6 atmospheres, 1-minute duration (Figure 3A). Cineangiogram confirmed that the SVC was widely patent (Figure 3B). The gradient measured prior initiation of the procedure was 20 mmHg. The post-serial balloon inflation gradient was reduced to 1 mmHg.

His edema and neck vein distention completely resolved after the procedure. Seven months later, the patient presented with angina. During coronary catheterization, a venogram was done and showed a patent SVC.

**Figure 4:**
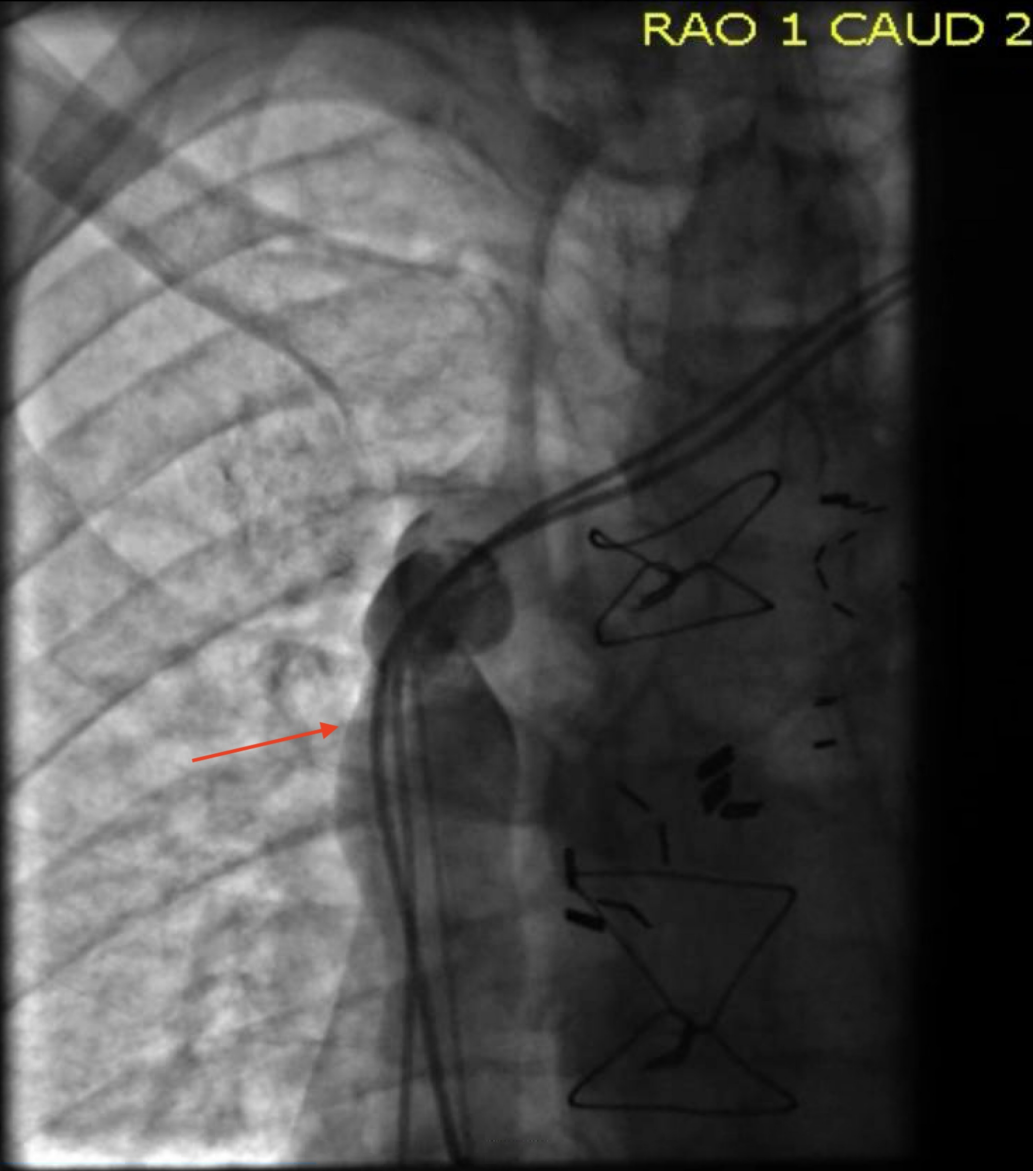
A venogram confirmed a patent superior vena cava (SVC) (red arrow)

The patient continued to be without symptoms of SVC obstruction until he passed away seven years later. This was significantly longer compared to previous studies. 

## Discussion

**Figure 5:**
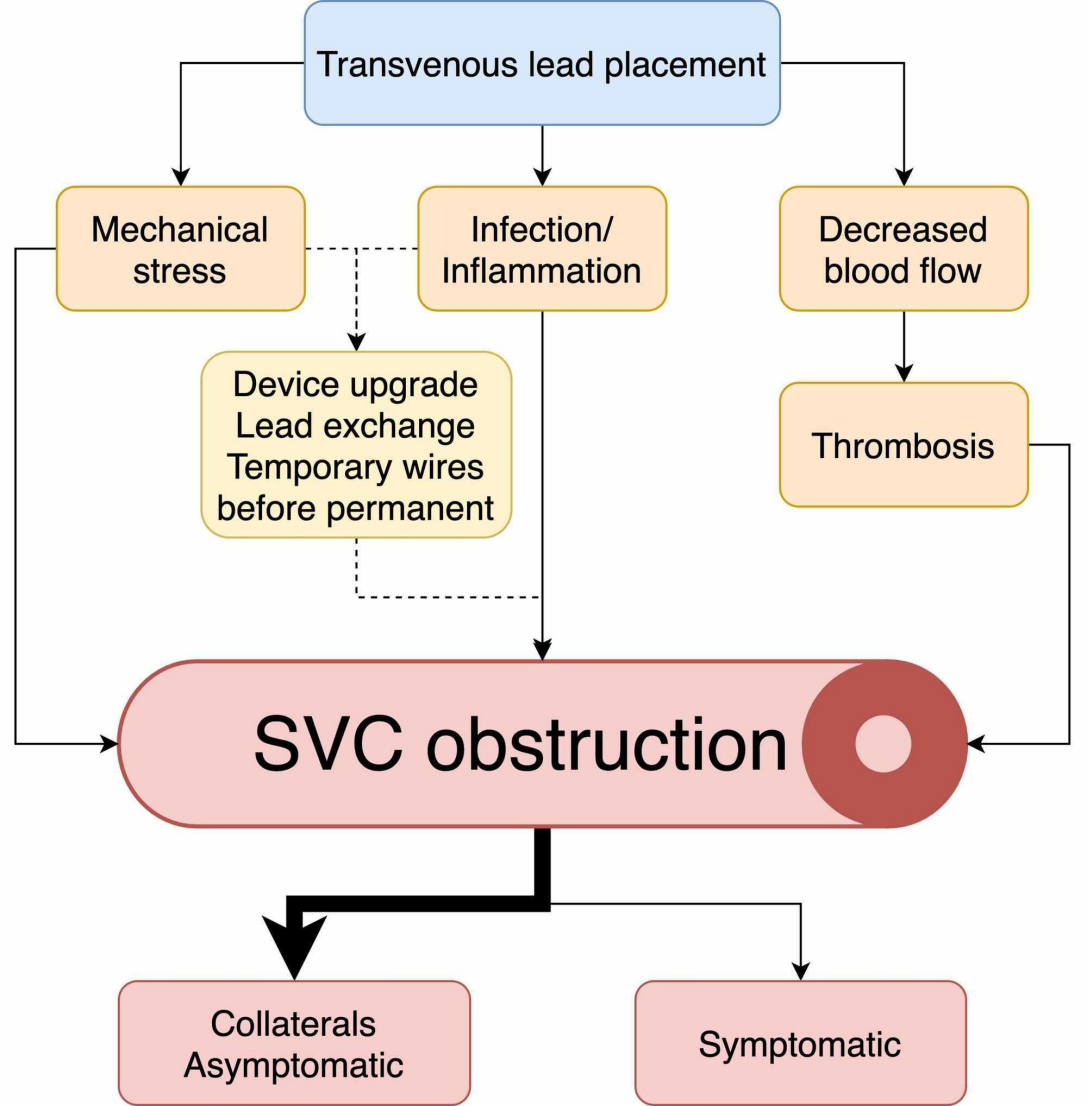
Pathogenesis of superior vena cava (SVC) obstruction in transvenous lead placement

SVC syndrome is a reflection of the evolution of medicine. The first published case described SVC syndrome in a patient with syphilis. For years, syphilis and tuberculosis remained the main causes of SVC syndrome until antibiotics were discovered, which almost eliminated infectious etiologies. This led to malignancies being the most common reason for SVC obstruction [1]. Medicine continued to evolve and incidences of SVC syndrome have been falling dramatically throughout the 20th century. However, another medical breakthrough is bringing SVC syndrome back in the modern era. Today, up to 40% of patients diagnosed with SVC syndrome do not have a malignancy causing the obstruction. Instead, they have SVC syndrome of benign etiology, such as pacemaker wires [2]. Studies have shown that at least a quarter of patients with transvenous leads develop some degree of SVC obstruction [3-4]. The mechanism of the SVC obstruction after lead placement is not fully understood and is likely multifactorial. It has not been confirmed that the number of leads within the vein is associated with an increased rate of occlusion. However, a transvenous lead is a foreign body that causes mechanical stress on the vessel wall, inflammation, and infection which results in scarring and fibrosis [5]. Recent studies also showed that lead placement results in low blood flow between the lead and the vessel wall, predisposing these patients to thrombosis [6]. These factors lead to the narrowing of the SVC (Figure 5). However, not all patients will develop clinical symptoms, even with significant SVC obstruction. The majority of the patients will remain asymptomatic due to the development of collaterals [7]. 

Management of patients with an SVC obstruction secondary to transvenous leads has transformed during the years. Historically, these patients were treated with anticoagulation or thrombolysis. Thrombolysis restored venous patency early, minimized damage to the vessel endothelium, and reduced the risk of long-term complications [8]. This strategy was not always helpful. In most patients, an obstruction developed over a period of months, or even years, and fibrosis played a major role in this process [8]. In this case, thrombolysis was not going to address the etiology of the obstruction. 

Surgery alone, such as removing the thrombus and fibrous tissue, is an effective option; however, it is invasive, associated with significant morbidity, and the rates of restenosis are no better when compared to angioplasty [7, 9]. Another surgical option would be to remove the implanted pacemaker leads, but the fibrotic attachments that develop between them and the venous, valvular, and cardiac structures are major obstacles [8]. Additionally, there is no data proving that the removal of the leads provides a patent pathway for blood flow.

Percutaneous transluminal balloon venoplasty may be successful in eliminating or reducing the venous stenosis to relieve the symptoms of stenosis [8]. 

Current recommendations state that transvenous leads should be removed in patients with SVC syndrome [10]. However, that could be associated with multiple complications including, but not limited to, death, intimal flaps, thrombosis, and possible restenosis [11]. In addition, a recent review and case series have shown that performing a percutaneous intervention with transvenous leads still in the SVC is not associated with significant complications and has good outcomes at one to two years [9, 12]. However, the data regarding long-term outcomes is worse. A retrospective analysis of percutaneous transluminal angioplasty with central vein occlusion (n = 59, 53 of which had an indwelling catheter or leads) showed a failure rate of 75% by 33.8 months. This was unlike our patient, who remained symptom-free for more than seven years without evidence of SVC obstruction [8].

Combined therapy, such as balloon angioplasty following thrombolytic therapy, may have several advantages: (1) reduction in fibrotic stenosis, thus, reducing the risk of rebound stenosis, (2) mechanical removal of the thrombus, potentially decreasing the duration and dosage of thrombolytic therapy, and (3) the large initial lumen created may lead to a faster resolution of signs and symptoms caused by the obstruction [7]. Thus, percutaneous intervention is becoming the most widely used treatment modality in these patients, making surgery a last resort option when obstruction cannot be treated endovascularly.

## Conclusions

Herein, we presented a patient with a severe SVC obstruction secondary to pacemaker leads who was destined for the last resort of open thoracotomy because the lesion couldn’t be crossed using the standard techniques. As mentioned above, angioplasty is non-inferior to surgery in outcomes. However, in order to perform a successful venoplasty in these patients, the interventionalist must be familiar with a variety of techniques to cross the lesion. To our knowledge, this is the first case to show the long-term success of a percutaneous venoplasty in a patient with an SVC obstruction secondary to pacemaker wires. Our approach can help achieve long-term success and avoid open thoracotomy associated with high morbidity. 
